# The Association between Water Consumption and Hyperuricemia and Its Relation with Early Arterial Aging in Middle-Aged Lithuanian Metabolic Patients

**DOI:** 10.3390/nu15030723

**Published:** 2023-01-31

**Authors:** Alma Čypienė, Silvija Gimžauskaitė, Egidija Rinkūnienė, Eugenijus Jasiūnas, Rita Rugienė, Edita Kazėnaitė, Ligita Ryliškytė, Jolita Badarienė

**Affiliations:** 1 State Research Institute Centre for Innovative Medicine, 08406 Vilnius, Lithuania; 2 Faculty of Medicine, Vilnius University, 03101 Vilnius, Lithuania; 3 Center of Informatics and Development, Vilnius University Hospital Santaros Klinikos, 08661 Vilnius, Lithuania

**Keywords:** hyperuricemia, serum uric acid, water intake, carotid-femoral pulse wave velocity, cardiovascular disease, metabolic syndrome, risk factor, arterial stiffness

## Abstract

Background: Hyperuricemia is well-known as an independent risk factor for the development of hypertension, metabolic syndrome, and cardiovascular disease. Water is essential to most bodily functions, and its consumption rates appear to decline with age. The aim was to evaluate the influence of water intake on early vascular aging in metabolic middle-aged patients with hyperuricemia. Materials and Methods: The study included 241 men aged 40–55 years and 420 women aged 50–65 years from the Lithuanian High Cardiovascular Risk (LitHiR) primary prevention program. Anthropometric characteristics, blood pressure, laboratory testing, and the specialized nutrition profile questionnaire were evaluated. Carotid-femoral pulse wave velocity (cfPWV), assessed using applanation tonometry, was evaluated as an early vascular aging parameter in patients with hyperuricemia and with normal serum uric acid (sUA) levels. Results: 72.6% of men and 83.1% of women drink insufficient amounts of water (less than 1.5 L per day). However, our results showed statistically significant relationships only among a group of women. The women in the hyperuricemic group had a higher cfPWV than women with normal sUA levels. In hyperuricemic women, drinking less than 0.5 L per day in combination with other risk factors, such as age, increasing fasting glucose, and systolic blood pressure, was statistically significantly associated with an increased cfPWV (R^2^ = 0.45, Adj. R^2^ = 0.42, *p* < 0.001). Conclusion: Drinking an insufficient amount of water daily is associated with increased arterial stiffness and has a negative effect on vascular health in metabolic women with hyperuricemia.

## 1. Introduction

Hyperuricemia is defined as an elevated serum uric acid (sUA) level in the blood and is associated with purine metabolism and urate excretion through the kidneys and gastrointestinal tract [1]. Normal sUA levels are considered to be 210–430 μmol/L in men and 155–360 μmol/L in women. Hyperuricemia often manifests as gout, which is the most frequent form of arthritic disease, although more than half of the patients with hyperuricemia never develop clinical symptoms [2,3].

Asymptomatic hyperuricemia is now well-known as an independent risk factor that simultaneously interacts with many traditional cardiovascular (CV) risk factors in contributing to the development of cardiovascular diseases (CVD) [4]. Chronic hyperuricemia is correlated with clinical disorders such as hypertension, obesity, dyslipidaemia, insulin resistance, and elevated glucose levels, which together can be defined as the metabolic syndrome (MetS) [5]. Hyperuricemia is also considered a manifestation or adverse effect of MetS [6]. There is evidence from several cross-sectional and prospective studies that hyperuricemia is associated with a higher risk of developing hypertension, while 25–50% of hypertensive patients have already increased levels of sUA [7].

MetS and hyperuricemia are associated with unhealthy dietary habits and a sedentary lifestyle [8]. For many ages, gout was associated with the wealthy, who could afford to eat purine-rich foods at that time [9]. Although there are several studies analyzing the effects of different food products, diet types, or alcohol consumption, little is known about the impact of water intake on hyperuricemia [10,11]. sUA is mainly excreted through the kidneys (70%) and normal kidney function depends on optimal hydration status [12,13]. There is also evidence that dehydration caused by exercise-induced excessive sweating or sauna bathing increases sUA levels [14].

Most people, including health practitioners, take drinking water for granted, while the majority of them fail to consume recommended daily amounts of water. The European Food Safety Authority recommends a fluid intake of 2.5 L per day for men and 2.0 L per day for women [15]. The European Society for Clinical Nutrition and Metabolism (ESPEN) exaggerates the difference in water consumption for people aged 65 years or older—2.0 L/day for men and 1.6 L/day for women [16]. It is recommended to drink at least 1.5 L of fluid per day, and anything less is considered inadequate [15]. Water consumption rates appear to decline with age, with middle-aged and elderly people consuming significantly lower amounts of water [17]. We hypothesize that water consumption habits may have an effect on hyperuricemia and vascular aging in middle-aged metabolic patients.

## 2. Materials and Methods

### 2.1. Study Population

A prospective study was carried out from January 2018 until November 2019 in the sub-department of preventive cardiology at VUH Santaros Klinikos. The study protocol was approved by the Vilnius Regional Biomedical Research Ethics Committee (No. 158200-18/4-1006-521).

The Lithuanian High Cardiovascular Risk (LitHiR) primary prevention program started in 2006 with a strong multifactorial focus on middle-aged high CV risk patients to prevent early atherosclerosis development. One of the many strategic objectives of this program is to assess lifestyle modifications, including unhealthy diet patterns, in primary prevention patients with MetS. The required data for this particular study were extracted from the data collected in the LitHiR program.

This study included women aged 50–65 years and men aged 40–55 years with diagnosed MetS, which was defined using National Cholesterol Education Program Adult Treatment Panel III (NCEP ATP III) modified criteria. Criteria included (a) waist circumference ≥102 cm for men and ≥88 cm for women; (b) serum triglycerides (TG) ≥1.7 mmol/L; (c) high-density lipoprotein (HDL) <1.03 mmol/L for men and <1.29 mmol/L for women; (d) blood pressure ≥130/≥85 mmHg or receiving antihypertensive treatment; (e) fasting plasma glucose ≥5.6 mmol/L. A diagnosis of MetS was established when ≥3 risk factors were present. The specific age range of patients differed for the reason that 60-year-old women have a similar risk for CVD as 50-year-old men [18].

The exclusion criteria for this study were previously diagnosed coronary artery disease, silent myocardial ischemia, transient ischemic attack, ischemic and hemorrhagic stroke, peripheral artery disease, oncological disorders, advanced kidney or hepatic failure, chronic or persistent arrhythmias, gout, severe psychiatric disorders, as well as pregnancy, drug addiction, and treatment with xanthine oxidase inhibitors.

### 2.2. Assessment of the Study Population

The written consent form was obtained before any research procedures started. The clinical examination was performed in a quiet room with a comfortable temperature (22–24 °C) in the mornings. Participants were asked to fast and refrain from drinking alcohol and caffeinated drinks for 12 h before the examination.

Physical measurements included height, weight, body mass index (BMI), waist circumference, and blood pressure. Blood pressure was measured using a manual sphygmomanometer (Riester precisa^®^ N Sphygmomanometer, Jungingen, Germany) following the 2018 European Society of Cardiology Guidelines for the Management of Arterial Hypertension [19]. Participants underwent venous blood sampling for the estimation of sUA, serum total cholesterol, HDL cholesterol, low-density lipoprotein (LDL) cholesterol, TG, serum creatinine, and high-sensitivity C-reactive protein (hs-CRP) levels. Blood samples were analyzed using the “Abbott Architect ci8200 PLUS” (Abbott Laboratories, Chicago, IL, USA) analyzer. Hyperuricemia was diagnosed when the sUA levels in the blood serum exceeded 357 µmol/L in women and 428 µmol/L in men. All patients were divided into two groups according to the presence of hyperuricemia.

For the assessment of arterial stiffness, carotid and femoral pulse waves were evaluated in the supine position in a non-invasive way by using the applanation tonometry technique (Sphygmocor v.7.01, AtCor Medical Pty. Ltd. 1999–2002, Sydney, Australia) while simultaneously recording an electrocardiogram (ECG). Pulse wave velocity (PWV) was determined by measuring the carotid-to-femoral (cfPWV) pulse wave transit time. The mean blood pressure (MBP) was calculated automatically using the formula (MBP = 1/3 systolic blood pressure + 2/3 diastolic blood pressure).

The specialized questionnaire contained 14 questions seeking information about the respondent’s place of residence (rural, urban, other), education (primary, secondary, university, doctorate), marital status (single, married, divorced, widower/widow), use of drugs (antihypertensive, statins, other drugs, no treatment), and smoking status. Participants were asked to mark how often they eat meat, dairy, and fish products. The options of frequency varied from 30 times per month to never/seldom. In addition to the nutrition assessment, the participants were asked about their water consumption per day and alcohol consumption in units per week. In this particular study, we emphasized daily water intake. The options were >1.5, 1–1.5, 0.5–1, and <0.5 L per day.

Efforts were made to repeatedly contact the participants, whose given information was ambiguous.

### 2.3. Statistical Analysis

Statistical analysis was performed using the R statistical software package V 4.0.2 (^©^The R Foundation for Statistical Computing), RStudio V 1.3.959 (^©^2009–2020 RStudio, PBC), IBM SPSS Statistics V.23, and G*Power V.3.1.9.4 (Universität Düsseldorf, Düsseldorf, Germany).

Interval and ratio variables were described by medians, first quartiles (Q1), and third quartiles (Q3). Shapiro-Wilk and Kolmogorov-Smirnov (K–S) tests were used to check the data for normality. Nominal and ordinal variables were characterized by frequencies and percentages across the corresponding subset of the sample.

We created models based on linear regression equations to assess the statistically significant influence of relevant independent variables on the dependent variable. To test for heteroscedasticity in a linear regression model, we used the Breusch-Pagan test. To assess the statistically significant difference among the independent groups, Pearson’s chi-squared test was used.

Spearman’s correlation coefficient (rS) was used to measure the size of the effect between interval variables. We assumed that the effect size was low if 0.1 ≤ rS < 0.3, moderate if 0.3 ≤ rS < 0.5, and large if rS ≥ 0.5.

The omega squared partial (ω2p) effect size was used to measure the effect size between normally distributed interval variables, and the rank epsilon squared ordinal (ε2ordinal) effect size was used to evaluate the effect size between interval variables, unsatisfying the condition for normal distribution. We assumed that the effect size was small if 0.01 ≤ ω2p (ε2ordinal) < 0.06, moderate if 0.06 ≤ ω2p (ε2ordinal) < 0.14, and large if ω2p (ε2ordinal) ≥ 0.14.

The relationships between variables were considered statistically significant when the *p*-value was less than 0.05 (*p* < 0.05) and a statistical test power of 1-ß was equal to 0.95 (1-ß = 0.95).

## 3. Results

A total of 661 patients were examined in this cross-sectional study. The study sample consisted of 241 men aged 40–55 years and 420 women aged 50–65 years. Men and women were divided into two groups with normal and increased sUA levels (171/70 in the male group and 276/144 in the female group, respectively). The median age in men/women was 46/58 years in a group with increased sUA and 48/58 years in a group with normal sUA levels.

There was a statistically significant difference in BMI, waist circumference, SBP, hsCRP, and creatinine levels between patients with normal and increased sUA levels. However, there was no difference in LDL cholesterol between study subjects. A more detailed analysis of the main clinical characteristics between groups is shown in Table 1.

We also compared both groups of men and women by their education, place of residence (rural, urban, other), marital status (single, married, divorced, widower/widow), use of drugs (antihypertensive, statins, other drugs, no treatment), and smoking status; however, our results showed no statistically significant difference between groups with increased and normal sUA levels in both genders.

The women in the hyperuricemic group had a higher cfPWV than women with normal sUA levels. There was no significant difference in cfPWV among men, although MBP was significantly higher in men with increased sUA levels (Table 2).

In this study, from a given nutrition profile questionnaire, we extracted data on daily water consumption only. The great majority of patients reported insufficient amounts of daily water consumption (less than 1.5 L per day) in both normal and increased sUA groups. Most often, participants have 1–1.5 L of water per day (42.7%), while only slightly more than one-fifth of study subjects (21.6%) drink more than 1.5 L per day. There was no statistically significant difference between water intake amounts in both groups of men and women with increased and normal sUA levels (Table 3).

**Figure 1:**
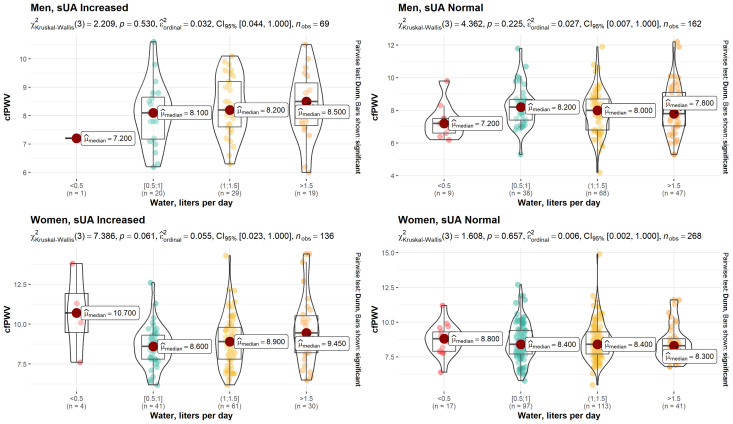
Dependence between water intake habits and cfPWV in patients with normal and increased sUA levels.

Analyzing the relationship between water intake habits and cfPWV, we determined a weak to moderately strong (ε2ordinal = 0.05) dependency (*p* = 0.006) in a subgroup of women with increased sUA (Figure 1). There were no statistically significant results in a subgroup of women with normal sUA levels or in a group of men with and without hyperuricemia.

**Figure 2:**
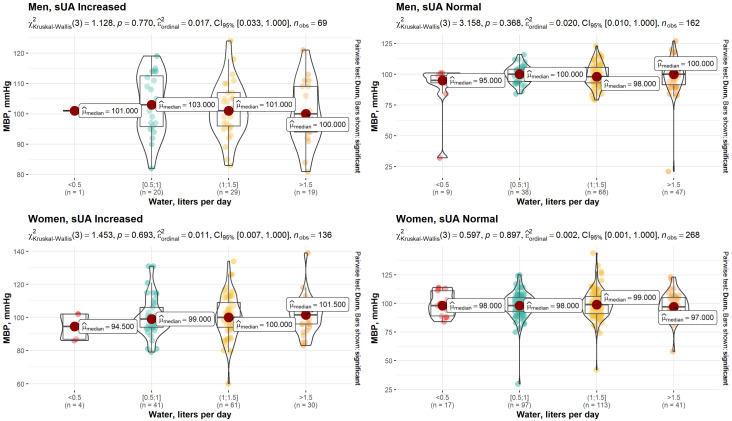
Dependence between water intake habits and MBP in patients with normal and increased sUA levels.

However, no statistically significant relationship between water intake habits and MBP was found in patients with normal and increased sUA levels (Figure 2).

**Figure 3:**
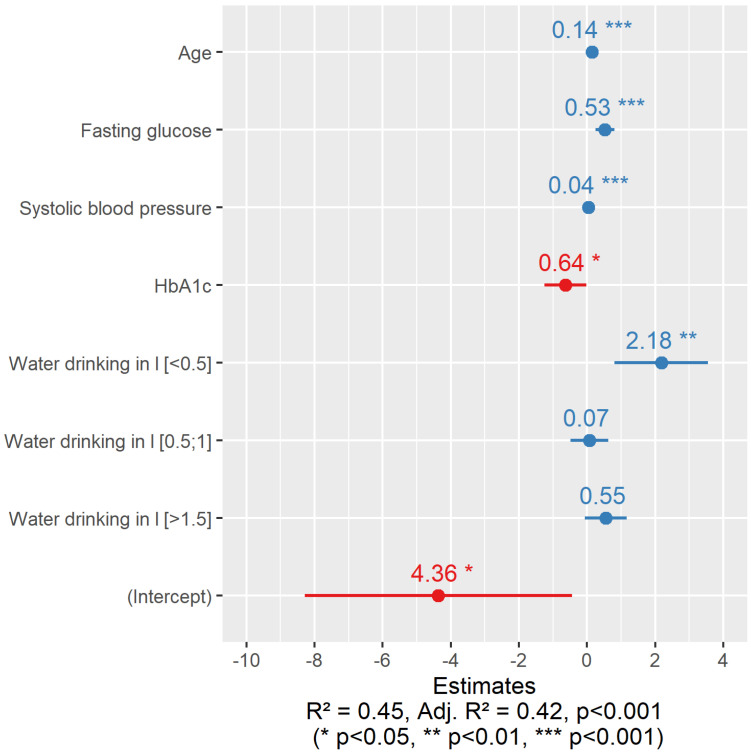
Coefficients of the linear logistic regression for cfPWV in women with increased sUA levels (N = 131). The positive values of the coefficients are presented in blue, and the negative values of the coefficients are presented in red.

As long as our study showed no statistically significant relationship on cfPVW in a group of men, we further highly focused only on a group of female patients. To evaluate the influence of certain risk factors on cfPWV, we constructed linear regression equations including all the indicators presented in Table 1 and Table 2. After testing several types of regression equations, we found that the linear regression equation most accurately predicts cfPWV. Moreover, the histogram of cfPWV is not much different from the bell shape. After optimization of the regression equations, we obtained that in a group of women with increased sUA, drinking less than 0.5 L of water per day in combination with other risk factors was statistically significantly associated with increased cfPWV (2.18) (Figure 3).

## 4. Discussion

In this cross-sectional study, the intent was to examine the influence of daily water consumption on one of the early arterial aging parameters—cfPWV—in middle-aged metabolic patients. Analysis of the data showed that cfPWV may differ between patients with elevated and normal sUA levels. Moreover, we found a sex-dependent relationship between sUA levels and vascular parameters of early arterial aging in the Lithuanian population. A similar significant correlation between elevated sUA levels and another arterial stiffness parameter—high brachial-ankle PWV—was observed in Korean women aged > 55 years, with no significant difference in men older than 45 years [20].

Aging, hypertension, obesity, and MetS contribute to arterial stiffness in both males and females. The effect of hyperuricemia on arterial stiffening and its gender differences are still controversial since numerous studies submit heterogeneous results. However, several factors could play a role here. Female subjects in our study were approximately 10 years older than male subjects, and age is a key factor for arterial stiffening [21,22]. The Baltimore Longitudinal Study of Aging showed that PWV increases over time, accelerating with each decade of life starting at age 40 [23].

We should also take into account a different hormonal background and the age-associated decline in sex hormone activity. According to ESC guidelines, early menopause is considered a risk factor for developing CVD [24]. Sex-related factors in developing MetS are associated with hyperandrogenism, insulin resistance, an increase in abdominal obesity, and HDL cholesterol reduction occurring after menopause [25]. Estrogen deficiency is associated with enhanced extracellular matrix remodeling, which affects the microstructure of vascular walls [26]. Estrogens also increase NO bioavailability in the endothelium in that way, decreasing vascular tone and thus slowing down the process of arterial stiffening [27]. Several studies showed that estrogen therapy reduced sUA levels and increased its excretion through the kidneys, proving the uricosuric effect of estrogens [28]. Several clinical conditions in women are also associated with arterial stiffening, such as polycystic ovary syndrome, endometriosis, and autoimmune disorders [29]. Considering hormonal status in men, lower serum testosterone levels are an independent negative predictor of the development of carotid artery stiffness, even after adjusting for risk factors such as age, pulse pressure, BMI, and total cholesterol [30].

The results of our analysis demonstrated a statistically significant difference in SBP between patients with elevated and normal sUA levels. Although sUA as a risk factor for CVD has been extensively investigated, little is known about the possible mechanisms by which hyperuricemia is related to hypertension and endothelial dysfunction. The endothelium, which forms the inner surface of all blood vessels in the body, is a hormonally active layer that normally secretes various vasoactive agents such as nitric oxide (NO), thromboxane 2, angiotensin II, etc. [31]. There is growing evidence that functional damage to the endothelium induces the development of systemic atherosclerosis, leading to the manifestations of CVD [32]. Endothelial dysfunction and hyperuricemia are closely associated and share many established CVD risk factors such as hypertension, diabetes, obesity, and therefore MetS. Recent clinical studies have mentioned that hyperuricemia might be causally related to endothelial dysfunction, although there are difficulties in evaluating the influence of sUA on the endothelium due to its close associations with traditional CVD risk factors [33].

It is considered that sUA decreases the production and bioavailability of NO and reduces local levels of L-arginine, which is required in NO synthesis, by reducing arginase activity, thereby causing endothelial dysfunction and hypertension. Other or complementary pathways by which hyperuricemia possibly lowers NO concentrations in endothelial cells, either by direct interaction with NO or by oxidative stress [34]. Studies in mice have shown that hyperuricemic animals given an inhibitor of uricase (an enzyme that degrades sUA to allantoin in mammals) develop hypertension and vascular disease, which are at least partially reversed by administration of L-arginine (a NO synthase substrate). Khosla et al. demonstrated a decrease in serum nitrites among rats with increased sUA levels and a trend toward increased SBP [35]. Choi et al. discovered that even at low or physiological concentrations, sUA can worsen endothelial dysfunction via insulin resistance [34].

Inadequate fluid intake induces the production of arginine vasopressin, which is associated with regulating normal water homeostasis and maintaining blood pressure. The results of the study by Lang et al. revealed that plasma vasopressin concentration was significantly higher among subjects who drink low amounts of water (less than 1.2 L/day) compared with those who drink more than 2 L/day. Studies in rats revealed that enhanced vasopressin and copeptin levels are related to higher fasting glucose levels, hyperinsulinemia, obesity, type 2 diabetes, and MetS [36].

A majority of our study subjects (72.6% of men and 83.1% of women) failed to meet EFSA and ESPEN recommendations to drink at least 1.5 L/day of water [15,16]. There were almost 5% of participants who drink inadequately low amounts of water (less than 0.5 L per day). The research conducted in the United States demonstrated that around 50% of all studied adults and even 83% of women and 95% of men in a subgroup of people older than 71 failed to meet the Institute of Medicine (IOM) Adequate Intake values [37]. Comparing the results of 13 different countries worldwide, it was reported that less than 50% of women and around 60% of men failed to meet the EFSA adequate intake values for water [38]. Our results revealed that women drink less water than men. Although other studies showed no significant difference in total fluid intake between men and women, they placed an emphasis on the social and educational aspects of water consumption rates, especially since individuals from developed countries tend to have a healthier lifestyle and, thus, a higher total fluid intake [38].

Insufficient water consumption and age-related changes in behavior make aging individuals susceptible to dehydration. Low fluid intake may be explained by the fact that the sensation of thirst decreases with age. Many other older adults avoid drinking, fearing urine incontinence during the night [17].

In our linear regression model, we obtained that insufficient daily water consumption, in combination with other risk factors, had a negative effect on cfPWV in women. Therefore, a patient’s individual risk likely represents a complex interplay between non-modifiable (e.g., age) and modifiable factors, such as BMI, lifestyle, or dietary habits.

Despite the lack of evidence, drinking water has shown numerous potential effects on metabolic and CV health. It was confirmed that slightly alkaline drinking water helps to keep blood vessels elastic and maintain blood pressure, which is beneficial for the CV system [39]. Alkalization of urine may have an effect on removing uric acid from the body [40]. The PREDIMED-Plus study investigated patients aged 55–75 years with MetS and revealed that increased drinking water was associated with a higher reduction in BMI and waist circumference [41]. Stookey et al. showed that water drinking in overweight and obese women was associated with improved weight loss even after adjustment for nutrition and physical activity [42]. The aforementioned effects seem to be an important confounder of our study results on cfPWV, as all study subjects were metabolic and the average BMI was more than 30 kg/m^2^.

Several studies emphasized considering the composition of drinking water. Trace elements (e.g., the mineral H_2_SiO_3_ (silicate)) in drinking water are associated with maintaining vascular health and preventing arteriosclerosis. Moderate levels of Ca and Mg help to avoid conditions such as CVD, hypertension, and osteoporosis and thereby promote longevity [43,44]. Studies in mice showed that water with higher dissolved oxygen concentrations reduced sUA levels and enhanced sUA metabolism [45]. Excessive fluoride concentrations in drinking water increased the prevalence of hyperuricemia among adolescents [46]. Consuming low-sodium or sodium bicarbonate-rich water significantly reduced mean arterial blood pressure in elderly normotensive patients [47]. In a mouse model, adding nitrite supplements to drinking water prevented vessel stiffness associated with aging [48].

Access to the sources of safe drinking water and its composition vary among different populations. Nowadays, about one billion people continue to suffer from poor access to clean water [49]. However, water drinking should be recognized as one of the most important modifiable lifestyle factors, which may be a valuable confounder in CV and especially metabolic health studies of the third millennium [50].

However, there were several potential limitations to this study. First of all, this was a cross-sectional study; thus, causal relationships between our results cannot be confirmed. We also collected data on early arterial aging parameters (cfPWV and MBP) at one point in time, without the longitudinal analysis over time. Further randomized, controlled, or cohort studies with larger sample sizes are still required. Second, study subjects were asked to report their daily water consumption rates, which are often underestimated and do not reflect the habitual water intake of an individual. We also were unable to estimate the amount of water obtained with different foods. As a result, failing to meet water intake recommendations on a given day may not accurately represent long-term water drinking status. Third, we were unable to control for environmental variables (geographical location, seasonality, etc.) that may have influenced water consumption rates. We underestimated the possible moderating effect of physical activity on arterial aging.

## 5. Conclusions

Although drinking water seems like a matter of course, most subjects indicated that their daily water intake was lower than recommended values. Drinking insufficient water is associated with increased arterial stiffness. Taken together, our results provide significant evidence that low fluid intake has a negative effect on vascular health among middle-aged metabolic women with hyperuricemia in a Lithuanian population, although there was no such effect in a group of middle-aged Lithuanian metabolic men. Further research is needed that would be aimed at increasing access to drinking water for the most vulnerable population groups, especially those with metabolic syndrome and hyperuricemia. Future studies should take into account the geographical availability of drinking water supplies among different populations and its effect on vascular health in metabolic patients.

## Figures and Tables

**Table 1:** Main clinical characteristics of patients.

Variable	Men (N = 241)			Women (N = 420)			Overall
	sUA Increased (N = 70)	sUA Normal (N = 171)	*p*-Value	sUA Increased (N = 144)	sUA Normal (N = 276)	*p*-Value	
Age, years			<0.05			0.51	
Median (Q1, Q3)	46.0 (43.0, 48.0)	48.0 (44.0, 51.0)		58.0 (54.0, 61.0)	58.0 (54.0, 61.0)		54.0 (49.0, 59.0)
BMI, kg/m^2^			<0.05			<0.05	
Median (Q1, Q3)	31.7 (30.1, 35.1)	30.5 (28.1, 32.8)		33.6 (30.1, 37.5)	30.5 (27.4, 33.6)		31.2 (28.4, 34.3)
Circumference of waist, cm			<0.001			0.06	
Median (Q1, Q3)	109 (104, 113)	105 (102, 110)		105 (97.0, 112)	97.0 (91.0, 105)		103 (95.0, 109)
SBP, mmHg			0.05			0.04	
Median (Q1, Q3)	139 (128, 148)	133 (126, 142)		137 (130, 146)	135 (123, 145)		135 (126, 145)
sUA, µmol/L			<0.05			<0.05	
Median (Q1, Q3)	481 (457, 520)	366 (328, 393)		397 (378, 447)	291 (259, 324)		352 (298, 404)
Median (Q1, Q3)	1.04 (0.913, 1.19)	1.05 (0.910, 1.25)		1.25 (1.09, 1.46)	1.35 (1.19, 1.56)		1.23 (1.04, 1.44)
LDL cholesterol, mmol/L			0.37			0.36	
Median (Q1, Q3)	3.81 (3.14, 4.28)	3.60 (2.92, 4.25)		3.67 (3.06, 4.36)	3.89 (3.01, 4.83)		3.74 (2.98, 4.49)
TG, mmol/L			0.44			<0.001	
Median (Q1, Q3)	2.39 (1.59, 2.83)	1.82 (1.26, 2.53)		1.79 (1.37, 2.43)	1.52 (1.07, 2.03)		1.73 (1.24, 2.39)
Fasting glucose, mmol/L			0.04			0.39	
Median (Q1, Q3)	5.98 (5.61, 6.51)	6.03 (5.63, 6.49)		6.13 (5.79, 6.94)	6.00 (5.66, 6.54)		6.06 (5.66, 6.60)
hs-CRP, mg/L			0.09			<0.001	
Median (Q1, Q3)	1.99 (1.16, 3.91)	1.46 (0.783, 2.35)		2.61 (1.15, 4.62)	1.46 (0.810, 2.76)		1.60 (0.880, 3.10)
Creatinine, μmol/L			<0.001			<0.001	
Median (Q1, Q3)	81.5 (77.0, 91.0)	77.0 (71.5, 84.0)		68.0 (62.0, 73.0)	65.0 (60.0, 70.0)		70.0 (63.0, 78.0)
Diabetes			0.92			0.07	
Yes	11.0 (15.7%)	26.0 (15.2%)		38.0 (26.4%)	52.0 (18.8%)		127 (19.2%)
No	59.0 (84.3%)	145 (84.8%)		106 (73.6%)	224 (81.2%)		534 (80.8%)

BMI—body mass index; TC—total cholesterol; LDL cholesterol—low-density lipoprotein; HDL cholesterol—high-density lipoprotein; TG—triglycerides; sUA—serum uric acid; hs-CRP—high-sensitivity C-reactive protein; and SBP—systolic blood pressure.

**Table 2:** cfPWV and MBP characteristics of patients.

Variable	Men (N = 241)			Women (N = 420)			Overall
	sUA Increased (N = 70)	sUA Normal (N = 171)	*p*-Value (Fisher’s)	sUA Increased (N = 144)	sUA Normal (N = 276)	*p*-Value (Fisher’s)	
cfPWV, m/s			0.14			0.004	
Median (Q1, Q3)	8.15 (7.53, 8.98)	8.00 (7.00, 8.75)		8.90 (7.80, 9.90)	8.40 (7.70, 9.33)		8.40 (7.60, 9.30)
MBP, mmHg			0.05			0.519	
Median (Q1, Q3)	101 (95.0, 109)	99.0 (93.0, 106)		99.0 (94.0, 106)	99.0 (91.8, 106)		99.0 (93.0, 106)

**Table 3:** Water intake habits of patients.

Variable	Men (N = 241)			Women (N = 420)			Overall
	sUA Increased	sUA Normal	*p*-Value (Fisher’s)	sUA Increased	sUA Normal	*p*-Value (Fisher’s)	
	(N = 70)	(N = 171)		(N = 144)	(N = 276)		
**Water, liters per day**			0.473			0.14	
<0.5	1 (1.45%)	9 (5.56%)		4 (2.94%)	17 (6.34%)		31 (4.88%)
(0.5–1)	20 (29.0%)	38 (23.5%)		41 (30.1%)	97 (36.2%)		196 (30.9%)
(1–1.5)	29 (42.0%)	68 (42.0%)		61 (44.9%)	113 (42.2%)		271 (42.7%)
>1.5	19 (27.5%)	47 (29.0%)		30 (22.1%)	41 (15.3%)		137 (21.6%)
Missing	1.00 (1.4%)	9 (5.3%)		8 (5.6%)	8 (2.9%)		26 (3.9%)

## Data Availability

The data presented in this study are available on request from the corresponding author.
